# Molecular alterations of driver genes in non-small cell lung cancer: from diagnostics to targeted therapy

**DOI:** 10.17179/excli2023-6122

**Published:** 2023-05-11

**Authors:** Anna Grodzka, Agnieszka Knopik-Skrocka, Katarzyna Kowalska, Pawel Kurzawa, Monika Krzyzaniak, Katarzyna Stencel, Maciej Bryl

**Affiliations:** 1 Department of Cell Biology, Faculty of Biology, Adam Mickiewicz University of Poznan, Poland; 2 Department of Oncological Pathology, University Clinical Hospital in Poznan, Poznan University of Medical Sciences, Poland; 3 Department of Clinical Pathology and Immunology, Poznan University of Medical Sciences, Poland; 4 Department of Clinical Oncology with the Subdepartment of Diurnal Chemotherapy, E. J. Zeyland Wielkopolska Center of Pulmonology and Thoracic Surgery, Poznan, Poland

**Keywords:** non-small cell lung cancer, driver genes, molecular alterations, targeted therapies

## Abstract

Lung cancer is the leading cause of cancer death all over the world. The majority (80-85 %) of lung cancer cases are classified as non-small cell lung cancer (NSCLC). Within NSCLC, adenocarcinoma (AC) and squamous cell carcinoma (SCC) are the most often recognized. The histological and immunohistochemical examination of NSCLC is a basic diagnostic tool, but insufficient for comprehensive therapeutic decisions. In some NSCLC patients, mainly adenocarcinoma, molecular alterations in driver genes, like *EGFR*, *KRAS*, *HER2*, *ALK*, *MET*, *BRAF*, *RET,* *ROS1, *and* NTRK* are recognized. The frequency of some of those changes is different depending on race, and between smokers and non-smokers. The molecular diagnostics of NSCLC using modern methods, like next-generation sequencing, is essential in estimating targeted, personalized therapy. In recent years, a breakthrough in understanding the importance of molecular studies for the precise treatment of NSCLC has been observed. Many new drugs were approved, including tyrosine kinase and immune checkpoint inhibitors. Clinical trials testing novel molecules like miRNAs and trials with CAR-T cells (chimeric antigen receptor - T cells) dedicated to NSCLC patients are ongoing.

## Introduction

**Figure 1:**
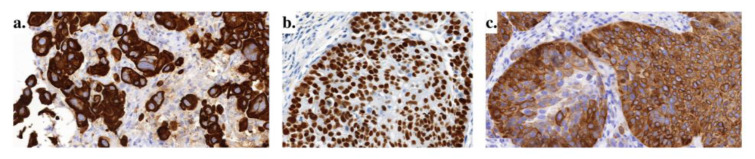
Positive IHC reactions in NSCLC. a) Adenocarcinoma - the tumor cells stain strongly for cytokeratin CK7, 400x; b) Squamous cell carcinoma - the tumor cells are positive for p40 and c) positive for cytokeratin CK5/6, 400x. The tissue samples were obtained from archived paraffin blocks collected in the Department of Oncological Pathology University Clinical Hospital in Poznań (photos and IHC reactions A. Grodzka, K. Kowalska, M. Krzyżaniak).

According to data from the World Health Organization from 2020, lung cancer is the second most frequently diagnosed cancer, with 12,2 % of new cases a year. Regarding mortality, lung cancer is responsible for 18,2 % of cancer-related deaths (Global Cancer Observatory https://gco.iarc.fr/, accessed 20 April 2023). The high death ratio may be caused by 70 % of lung cancer cases being diagnosed in an advanced or metastatic stage where radical therapy (surgery or radiotherapy) is impossible to implement (Lemjabbar-Alaoui et al., 2015[47]). According to clinical classification, lung cancer is divided into small cell lung cancer (SCLC) and non-small cell lung cancer (NSCLC), where the latter accounts for about 80-85 % of all lung cancer cases. The majority of NSCLC morphological recognition is based on a small amount of tissue (fine-needle aspiration or bronchoscopic biopsies) and two main subtypes can be distinguished: adenocarcinoma (AC) and squamous cell carcinoma (SCC). ACC accounts for 40-50 %, and SCC about 20-30 % of NSCLC cases (Osmani et al., 2018[63]; Zheng, 2016[100]). ACC and SCC show positive immunohistochemical reactions (IHC) for specific markers (Table 1(Tab. 1); Reference in Table 1: Zheng, 2016[100]). These IHC reactions demonstrate high sensitivity and specificity (Kriegsmann et al., 2019[46]). Figure 1(Fig. 1) shows the results of some IHC reactions in ACC and SCC. Some NSCLC cases could not be diagnosed only with regard to morphological features and remained NSCLC-NOS (NOS- not otherwise specified). IHC investigation of these samples leads to a more precise diagnosis (Righi et al., 2014[75]).

IHC is also used to examine PD-L1 status in NSCLC patients. This protein is a ligand of one of the immune checkpoints. Based on Keynote 001 lung cohort results, two cutoff points (1 % and 50 %) were established (Garon et al., 2015[28]). The higher one (50 %) was recognized as the predictive marker of first Pembrolizumab and further Atezolizumab and Cemiplimab monotherapy (Reck et al., 2016[73]). Many evaluations were performed to assess PD-L1 prevalence. In the Polish study, IHC PD-L1 expression in ≥1 % of cancer cells was observed in 32,5 % of NSCLC patients (Pawelczyk et al., 2019[65]). High expression in ≥50 % of tumor cells were detected in approximately 23 to 28 % of advanced NSCLC in the Pembrolizumab registration trial (Reck et al., 2016[73]). The data from large clinical trials are summarized in Table 2(Tab. 2) (References in Table 2: Gandhi et al., 2018[26]; Johnson et al., 2023[40]; Jotte et al., 2020[42]; Pawelczyk et al., 2019[65]; Paz-Ares et al., 2018[66]; Reck et al., 2021[72]).

For example, studies in which high PD-L1 expression (50-70 %) was reported were conducted on the Asian population. Unfortunately, clinical series that correlated PD-L1 expression with clinicopathologic and/or molecular variables and/or survival have reported conflicting results. Probably, not only differences in ethnicity and/or histologic types but also differences in the PD-L1 IHC method can be responsible for this (Mino-Kenudson, 2016[59]).

NSCLC patients, compared to SCLC, show a higher frequency of driver gene alterations (Vollbrecht et al., 2015[88]). It is known that their accumulation successively increases uncontrolled cell proliferation (Tomasetti et al., 2017[84]). Regarding the complex genetics of NSCLC, primary attention is paid to molecular diagnostics. The alterations in *EGFR* and *KRAS* genes are the most frequently observed in NSCLC. *ALK*, *ROS1*, *HER2*, *BRAF*, *MET*, and *RET* or *NRTK* are other driver genes involved in NSCLC oncogenesis. Molecular alterations within these genes are mainly point mutations, amplifications, chromosome rearrangement, or protein overexpression (Ren et al., 2022[74]; Zhu et al., 2017[102]). 

## Molecular Diagnostics in NSCLC and the Driver Genes

Many methods, like PCR, qPCR, RT-PCR, FISH, and NGS, are involved in assessing NSCLC molecular alterations (Table 3(Tab. 3); References in Table 3: König et al., 2021[45]; Manea et al., 2022[56]; Osta et al., 2020[64]; Ren et al., 2022[74]; Zhu et al., 2017[102]). The role of IHC is limited only to the evaluation of protein overexpression or expression of pathologic one. FISH is helpful mainly for detecting chromosome rearrangements. PCR methods identify chosen DNA sequences. NGS enables the simultaneous evaluation of specific genes using a cancer gene panel. This method can also be used for transcriptomic and epigenetics analysis (Dong et al., 2019[20]). However, according to an international survey conducted by the International Association for the Study of Lung Cancer (IASLC), the adoption of molecular testing for lung cancer is suboptimal. Most patients with molecular alterations were only tested for EGFR and ALK. Unfortunately, there are still many limitations, like testing cost and access (Smeltzer et al., 2020[77]). 

The material for genetic studies is usually the tissue sample or the cell aspirate from a biopsy, but if the sample is insufficient, or the risk of biopsy is too high, a liquid biopsy emerges as a reasonable option (Smolle et al., 2021[78]). DNA in the blood comes from circulating tumor cells, exosomes, or circulating cell-free material (Ansari et al., 2016[3]; Chen and Zhao, 2019[11]). Liquid biopsy is not only a diagnosis of genetic alterations, but it also has the potential to monitor the response to the treatment and the development of acquired resistance (new driver mutations in cancer cells).

About 50 % of NSCLC patients do not show any known molecular alterations (Stencel et al., 2021[81]). The driver mutations are recognized mainly in AC cases (Joshi et al., 2021[41]). Only 4-5.8 % of SCC patients in Asia show *EGFR* mutations and 1-1.7 % *KRAS* molecular changes (Gou and Wu, 2014[29]; Joshi et al., 2021[41]). For AC, significant differences in the frequency of *EGFR* and *KRAS* genetic alterations are observed among Caucasian and East Asian populations (Joshi et al., 2021[41]). *KRAS* mutations are the most common in the Caucasian group (25-50 %), while *EGFR* gene mutations dominate in East Asians (27-62 %).

**Figure 2:**
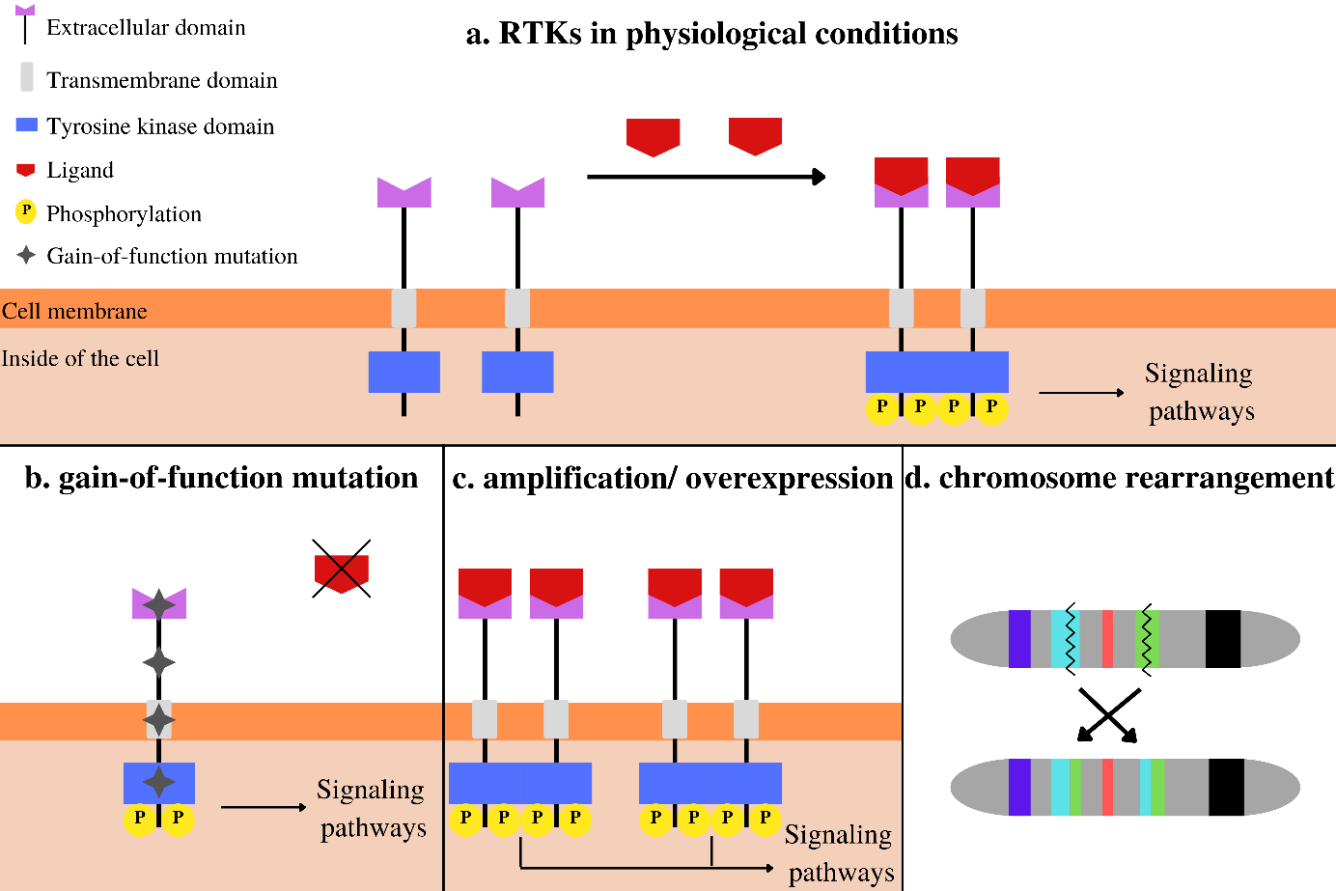
Schematic illustration of RTKs action and their changes; (a) physiological conditions; (b) gain-of-function mutations; (c) gene amplification/receptor overexpression; (d) chromosomal rearrangement (Du and Lovly; 2018; Gandhi et al., 2015; Noor et al., 2020).

Many molecular driver alterations in NSCLC involve receptor tyrosine kinases (RTKs) genes. They are proteins responsible for controlling a wide range of biological processes by involvement in cell-to-cell communication. For example, they regulate cell growth, differentiation, and metabolism and are associated with oncogenesis. The main components of RTKs are an extracellular ligand binding domain, a single transmembrane helix, and an intracellular region - a tyrosine kinase domain. Its activation is a result of ligand binding and receptor dimerization and/or oligomerization (Figure 2a(Fig. 2); References in Figure 2: Du and Lovly; 2018[23]; Gandhi et al., 2015[27]; Noor et al., 2020[62]). It tends to conformational changes and autophosphorylation. The next step is an activation of cascades of other intracellular proteins of signaling pathways. Under normal physiological conditions, the number of RTKs and their activity is strictly regulated. Their dysregulation in cancer cells can be the result of different molecular alterations, like activation by gain-of-function mutations (Figure 2b(Fig. 2)), RTKs proteins overexpression- following gene amplification (Figure 2c(Fig. 2)), or chromosomal rearrangements (Figure 2d(Fig. 2)) (Du and Lovly, 2018[23]).

## EGFR

EGFR (ErbB1) is a transmembrane tyrosine kinase receptor, which belongs, like HER2/neu (ERBB2), HER3 (ERBB3), and HER-4 (ERBB4), to the ErbB family (Hsu et al., 2019[31]). *EGFR* gene mutations in cancer cells have been found in 2004 (Lynch et al., 2004[55]). They were the first targetable and predictive oncogenic driver alteration discovered in lung cancer. A deletion in exon 19 (del19) and point mutation in exon 21 of the *EGFR* gene (L858R) is the most frequent (König et al., 2021[45]). It results in receptor hyperactivation, and then cell proliferation is observed. *EGFR* mutations have significantly higher rates in never-smokers, east Asians race, females' gender, and younger age (Couraud et al., 2012[15]). The study of Warth et al. (2014[90]) shows that this alteration is almost doubled in women (21.7 % women vs 11.7 % men). EGFR IHC is useless as a diagnostic tool because targetable *EGFR* mutations do not influence the extent of EGFR expression at the cell surface. That is why genotyping is a golden standard for *EGFR* (Yang et al., 2022[96]).

NSCLC cell lines expressing mutant EGFRs show low expression of some negative regulators for EGFR (Yang et al., 2015[95]). One of them is tumor suppressor CD82, which is upregulated by wild type of *EGFR* but downregulated by mutant EGFRs. This change can be critical for elevated tumorigenic activity triggered by *EGFR* mutations. *EGFR* gene amplification and protein overexpression were also found in NSCLC patients but rather in SCC (Du and Lovly, 2018[23]; Hirsch et al., 2003[30]). Those alterations are not clinically meaningful*.*

## KRAS

KRAS is a member of the rat sarcoma oncogenes family (RAS). The *RAS* gene encodes a low molecular weight G protein with GTPase activity that acts as molecular signal transduction of cell growth and differentiation (Xie et al., 2021[92]). *KRAS* mutation is one of the most frequent alterations in human cancers and NSCLC (König et al., 2021[45]; De Maglio et al., 2022[18]). It leads to the constitutive activation of the KRAS protein and the subsequent signal transduction (De Maglio et al., 2022[18]). The majority of mutations are located in codon 12, and point mutation variant G12C (glycine replaced by cysteine at codon 12) is the most frequent (Michelotti et al., 2022[58]). *KRAS* mutation is substantially associated with smoking status. Most patients with NSCLC harboring *KRAS* G12C mutation were current (40.7 %) or former (50 %) smokers. This mutation has prognostic value (Finn et al., 2021[25]). It is significantly associated with poorer prognosis. The risk of death is higher for *KRAS* G12C mutated patients compared with *KRAS*-nonmutated or *KRAS *other mutations by 32 % and 39 %, respectively.

## HER2

Human epidermal growth factor receptor 2 (HER2 or ErbB2) is a RTK that belongs to the same family as EGFR. These receptors consist of a ligand-binding extracellular domain and an intracellular tyrosine kinase domain. Ligand binding induces a homo- or heterodimerization with other family members. HER2 heterodimerizes with other HER receptors and entails activation of downstream signaling through PI3K/AKT and RAS/MAP/ MEK pathways (Pillai et al., 2017[68]). There are three types of molecular alterations of *HER2* in NSCLC: activating mutations, gene amplification, and protein overexpression (Ren et al., 2022[74]). In breast cancer, HER2 overexpression often occurs concurrently with amplification. In lung cancer, significant correlation exists between *HER2* gene copy number, and protein overexpression. *HER2* amplification and *HER2* mutations are mutually exclusive (Uy et al., 2022[86]). 

HER2 overexpression in NSCLC is a complex phenomenon with distinct molecular features making this alteration a weak biomarker in NSCLC. Its commonness is described with a wide range (Table 3(Tab. 3)), probably because of the lack of consensus on defining HER2 overexpression using IHC in NSCLC. The poor association between *HER2* amplification and HER2 overexpression in NSCLC, compared to breast cancer, is probably the reason for the low effectiveness of HER2-targeted therapies in NSCLC. Although the overlap between IHC 3+ staining and HER2 amplification was found, the IHC low/negative probes were FISH-positive (Yu et al., 2022[97]). Currently, only mutation is recognized as a valid biomarker for therapeutic decisions (Li et al., 2022[50]). Mutations are mainly found in females (62.4 %), never-smokers (60.4 %), and patients with AC (Mazières et al., 2016[57]). The rates of *HER2* alteration cases in NSCLC differ by country (Ren et al., 2022[74]). For the USA, mutations are observed in 3 % of cases, amplification in 3 %, and overexpression in 0 %, whereas in China, mutations are equal to 4.8 %, amplification to 15 %. Interestingly, *HER2* gene alterations can coexist with TP53 mutation (Xu et al., 2020[93]).

## ALK

ALK in physiological conditions is expressed in neural tissue, the small intestine, but it is not present in healthy lungs (König et al., 2021[45]). ALK regulates signaling pathways shared with other RTKs, like MAPK, PI3K-AKT, and JAK-STAT. Several fusions of the *ALK* gene were discovered. The most common is rearrangement with echinoderm microtubule-associated protein-like 4-four genes (*EML4*). Both are located on chromosome 2 but *ALK *at P23 and *EML4* at P21. More than 21 forms of *ALK-EML4* have been reported (Dong et al., 2019[20]; Liu et al., 2019[54]). The fusion is a consequence of inversion on the short arm of chromosome 2. EML4 is joined to the intracellular tyrosine kinase domain of ALK, and thus it promotes dimerization and oligomerization, inducing constitutive activation of the ALK kinase (König et al., 2021[45]; Liu et al., 2019[54]). FISH is the golden standard for detecting *ALK* gene rearrangements (Dong et al., 2019[20]). However, because of a strong association between *ALK* gene rearrangement and ALK protein expression, IHC is also a good tool for pre-screening or preliminary tests (Yang et al., 2022[96]).

This molecular alteration, with a frequency of less than 10 % of NSCLC cases, is more frequent in non-smokers, younger age patients, and AC. For example, according to data from the European Thoracic Oncology Platform Lungscape iBiobank, 5.4 % of patients with NSCLC are *ALK*-positive, among them 79.2 % were AC (Letovanec et al., 2018[49]). Analysis of almost 20 thousand patients with NSCLC from the USA revealed that only 2.6 % of cases were *ALK*- positive, most at 18-44. Moreover, non-smokers had the most significant mutation rate, and *ALK *alteration was very rare if *EGFR*, *ROS1*, *KRAS,* or *BRAF* changes were present (Allen et al., 2020[2]).

## MET

*MET* proto-oncogene also encodes one of the RTKs. Hepatocyte growth factor as a ligand leads to receptor dimerization and autophosphorylation of tyrosine residues. Via signaling pathways, like MAPK or PI3K, cell proliferation, migration, invasion, angiogenesis, and the epithelial-to-mesenchymal transition can be activated (Drilon et al., 2017[21]). *MET* alterations found in NSCLC patients can include gene amplification, mutation, or protein overexpression leading to aberrant activation of downstream pathways (Michelotti et al., 2022[58]). *MET* exon 14 skipping mutation is observed in 2-4 % of cases, resulting in a decreased degradation of the MET protein and increased activation of downstream signaling pathways. *MET*ex14 mutation is recognized mainly in patients with AC (68.8 %), over 65 years old (79 %), and females (60.4 %) (Schrock et al., 2016[76]; Yang et al. 2022[96]). This molecular alteration can be identified by DNA-based sequencing in most cases. However, some *MET*ex14 skipping mutations might be caused by alterations in deep intronic regions which could not be detected by assay limited to exonic and canonical splice site sequences. *MET*ex14 skipping mutations usually coexist with overexpression of this gene but MET IHC has minimal utility as a diagnostic tool for *MET*ex14 skipping alterations (Yang et al., 2022[96]). *MET* changes detected by liquid biopsy occur in more patients, than *MET* alterations found in tissue (Ikeda et al., 2018[34]). *MET* alterations, mainly gene amplification, strongly impact the therapy with RTK inhibitors in *EGFR*-mutant NSCLC patients (Zhang et al., 2019[99]).

## BRAF

BRAF, full name v-Raf murine sarcoma viral oncogene homolog B is a serine/threonine kinase, a part of the MAP/ERK signaling pathway. This pathway might be deregulated due to activating point mutation of the *BRAF* gene. *BRAF* mutations in NSCLC are rare (Table 3(Tab. 3)) and commonly occur in never-smokers, women with AC. Recently three classes of *BRAF*V600 mutations have been distinguished: class I- including RAS-independent kinase-activating V600 functioning as monomers; class II- RAS-independent kinase-activating nonV600 dimers; class III- RAS-dependent kinase-inactivating nonV600 heterodimers (König et al., 2021[45], Yan et al., 2022[94]). 50-80 % of *BRAF* mutations in NSCLC are nonV600 and belong to class II or III (Bracht et al., 2019[7]; Yan et al., 2022[94]). *BRAF* mutations in lung cancer can coexist with *EGFR *and* KRAS* molecular alterations (Li et al., 2014[51]). It is not common to analyze *BRAF* gene mutation separately, but it is encouraged to include it in the gene panel strategy (Yan et al., 2022[94]). 

## RET

Among NSCLC patients, *RET* gene alterations are infrequent (Table 3(Tab. 3)). The gene was discovered in 1985 (Takahashi et al., 1985[83]). This protooncogene on chromosome 10q11.2 encodes a transmembrane glycoprotein receptor tyrosine kinase with a ligand that belongs to the factors of the glial cell line-derived neurotrophic family (Choudhry and Drilon, 2020[13]). It can be activated by two mechanisms: *RET* fusions and *RET* point mutations (Osta et al., 2020[64]). Chromosomal rearrangement of *RET* can be the effect of the fusion of the 3`coding region for RET kinase domain on chromosome 10 with a 5` upstream partner gene with domain coiled-coil or *LIS1* homology (Choudhry and Drilon, 2020[13]). Intrachromosomal rearrangements are found in genes such as *KIF5B* (72 %) and *CCDC6* (23 %) (Osta et al., 2020[64]). Chimeric fusion proteins, produced by rearrangement, can cause ligand-independent constitutive activation of RET, promoting cancer cell proliferation (Drusbosky et al., 2021[22]). However, not all RET structural variants result in oncogenic fusion proteins because some are not associated with RET kinase fusions. Considering this, DNA- and RNA-based sequencing methods need to be used to discover the significance of particular structural variants (Yang et al., 2022[96]). *RET* fusions, discovered in 2012, are reported mainly in young, never smokers, with AC (Choudhry and Drilon, 2020[13]). 

## ROS1

ROS1 is a member of the insulin receptor family, and its extracellular domain is one of the largest among all human RTKs (Bubendorf et al., 2016[9]; König et al., 2021[45]). Its ligands in humans remain unknown, although, in studies with mice, chickens, and rats, ROS1 expression has been detected in the epithelial cells of the kidneys, male reproductive organs, small intestine, heart, and lungs. **ROS1** activates downstream oncogenic pathways, like STAT3, PI3K/AKT/mTOR, and RAS-MAPK/ERK, which controls cell proliferation (Zhu et al., 2015[101]). Similar to *ALK*, *ROS1* gene alteration in NSCLC is a chromosome rearrangement (Lin and Shaw, 2017[52]). The *ROS1* gene is located at q21 of chromosome 6, and rearrangement is mainly concentrated in exons 32-36 (Dong et al., 2019[20]). Fourteen fusion partners have been found: the most frequent is *CD74*. In contrast to the *ALK* gene, *ROS1* fusion partners do not provide dimerization domains that induce constitutive kinase activation (Lin and Shaw, 2017[52]). IHC might distinguish patients with *ROS1* rearrangements, but because of low specificity, the diagnosis must be confirmed by FISH (Yang et al., 2022[96]). The molecular alteration of *ROS1 *is relatively rare in NSCLC patients (only approximately 1 %), and it is detected mostly in AC (86 % of NSCLC cases with *ROS1* rearrangements). *ROS1* alterations are slightly more frequent among women (Cui et al., 2020[17]), and non-smokers (Song et al., 2017[80]). 

## NTRK

The neurotrophic tyrosine receptor kinase family (NTRK or TRK) comprises *NTRK1*, *NTRK2*, and *NTRK3* genes. They encode proteins of the tropomyosin receptor kinase (TRK) family, transmembrane receptor tyrosine kinases responsible for neuronal development (Manea et al., 2022[56]). Alterations of *NTRK* genes can be involved in carcinogenesis in neurogenic and non-neurogenic cells. NTRKs may become a part of fusion oncogenic proteins in different types of tumors but rarely in NSCLC (König et al., 2021[45]). NTRK1 and NTRK2 are preferentially expressed in SCC. It has been found that the presence of NTRK2 in SCC cases is correlated with a good prognosis (Liu et al., 2022[53]). Gene fusions affect different genomic rearrangements, but the 3' sequences of the *NTRK* gene are always fused to the 5' sequence of a fusion partner gene. It eventually leads to persistent activation of downstream signaling pathways involved in cell growth, proliferation, differentiation, survival, and apoptosis prevention (Liu et al., 2022[53]). Numerous fusion partners for the *NTRK* gene in NSCLC have been found: *MPRIP, CD74, QSTM1, TPR, IRF2BP2, BCL9, LMNA*, and *PHF20* (Liu et al., 2022[53]). The existence of NTRK fusions and other molecular alterations characteristic of NSCLC is mutually exclusive (Manea et al., 2022[56]). 

## Targeted Therapies in NSCLC and Perspectives

Driver mutation genes found in NSCLC patients are the excellent target in precise and personalized therapies with tyrosine kinase inhibitors (TKIs), immune checkpoint inhibitors (ICIs), or CAR-T cells. TKIs are the first targeted drugs for NSCLC patients (Michelotti et al., 2022[58]). During the last several years, ICIs were also approved for immunotherapy of NSCLC patients (Jiang et al., 2019[38]). Many clinical trials are focused on testing different drug combinations. Studies of CAR-T cell therapy applications in solid tumors, including NSCLC, are conducted. Also, epigenetic targets, like miRNAs, can be involved in developing new therapeutic strategies in NSCLC (Ahn et al., 2020[1]; Wang et al., 2019[89]). 

### Tyrosine kinase inhibitors (TKIs)

**Figure 3:**
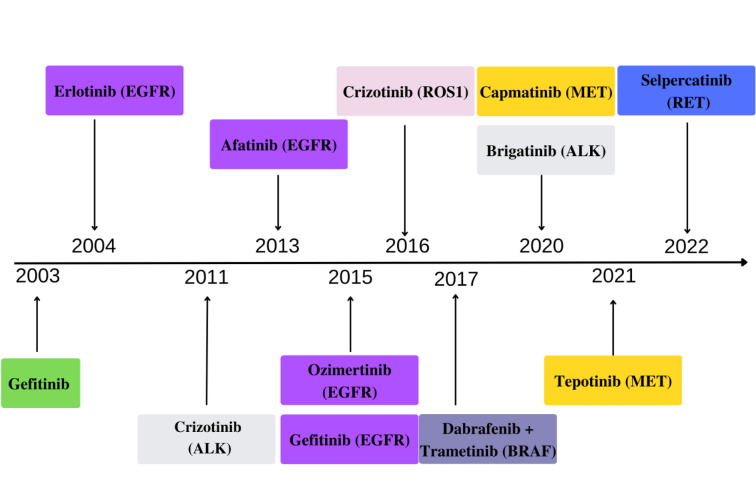
Timeline progress in NSCLC targeted therapy with FDA-approved TKIs (Cohen et al., 2003, Michelotti et al., 2022; U.S. Food & Drug Administration https://www.fda.gov/, accessed 20 April 2023, changed).

The first TKI approved by FDA for NSCLC patients was Gefitinib (Figure 3(Fig. 3); References in Figure 3: Cohen et al., 2003[14]; Michelotti et al., 2022[58]) as a treatment for patients with locally advanced or metastatic disease after failure of platinum-based and docetaxel chemotherapies (Cohen et al., 2003[14]), without indication of any gene mutation. The next was Erlotinib, whose mechanism of action was based on reverse binding to the cytoplasmic domain of EGFR as a target. During the following years, other TKIs were approved, including Afatinib and Gefitinib (EGFR mut). Osimertinib, approved in 2015, is the third-generation TKI against EGFR, both frontline and after treatment with first- or second-generation EGFR-TKIs (Zhang et al., 2019[99]). Crizotinib was the first-generation TKI for patients with *ALK* rearrangement. Second-generation ALK inhibitors are Ceritinib, Alectinib, and Brigatinib, followed by third-generation Lorlatinib (Michelotti et al., 2022[58]). *ROS1 *alterations are highly sensitive to Crizotinib and *BRAF*V600 to a combination of Dabrafenib with Trametinib. However, NSCLC patients with *BRAF* non-V600 mutations show much less sensitivity than V600 to these drugs, and novel BRAF kinase inhibitors are tested in clinical trials (Bracht et al., 2019[7]; Yan et al., 2022[94]). Over the last two years, Tepotinib (MET inhibitor) and Selpercatinib (RET inhibitor) were approved. The regular approval of Selpercatinib was in 2022. However, the first approval as an accelerated was based on the LIBRETTO-001 trial (NCT 3157128) in 2020 (Michelotti et al., 2022[58]). 

Targeted therapies with TKIs prolong the overall survival of NSCLC patients. However, during therapy, the acquired resistance to these inhibitors is developed. More than 50 % of the resistance to the first-generation of EGFR-TKIs results from *EGFR* exon 20 T790M mutations (Benedettini et al., 2010[6]). It was found that amplification of *MET* also can be responsible for acquired resistance to EGFR TKIs, e.g., Osimertinib. *MET* amplification is diagnosed in 5-22 % of *EGFR*-mutated NSCLC patients with acquired resistance to EGFR-TKIs (Michelotti et al., 2022[58]; Zhang et al., 2019[99]). These patients should be treated simultaneously with EGFR and MET inhibitors (Zhang et al., 2019[99]). *MET* amplification is not connected with *EGFR* exon 20 T790M mutation in some patients. According to data for the Korean NSCLC patients' group (Ji et al., 2013[37]), the coexistence of T790M mutation and *MET *amplification was found in 11.5 % of patients, and almost the same result was obtained for the group with only *MET* alteration. 

The mechanisms responsible for the development of acquired resistance to EGFR-TKIs can be divided into three main groups: 1) secondary mutations on *EGFR* or *EGFR* amplifications, 2) activation of new signaling pathways or different gene amplification (e.g. *MET*), and 3) phenotypic plasticity and epithelial-mesenchymal transition or transformation to small-cell lung cancer (Delahaye et al., 2022[19]). 

Concerning the contribution of MET in EGFR-TKI resistance, many clinical trials were conducted with molecules acting as inhibitors of MET. For example, Capmatinib was tested in GEOMETRY mono-1 and Tepotinib in VISION (Wolf et al., 2020[91]). The FDA approved these drugs in 2020 and 2021, respectively (Michelotti et al. 2022[58]). 

A driver oncogene *RET* can also be developed as a mechanism of acquired resistance to *EGFR *mutation during therapy with EGFR inhibitors. Most *RET* fusions involved in the resistance are *CCDC6* (58 %) and *NCOA4* (26 %) (Osta et al., 2020[64]). Selpercatinib and Pralsetinib are new RTKIs for *RET* fusion-positive NSCLC patients (Michelotti et al., 2022[58]). According to the results of clinical trials, Selpercatinib and Pralsetinib can be used as potent and selective inhibitors of *RET* fusions and mutations, irrespective of the tissue of origin (Drusbosky et al., 2021[22]). These RET-TKIs cross the blood-brain barrier and show tolerable toxicity profiles. It was described that acquired resistance to Selpercatinib can be developed during long-term RET inhibition. *RET*G810 mutations have been found in circulating tumor DNA and patient-xenograft model (Solomon et al., 2020[79]). However, the resistance can also be *RET*-independent, such as acquired *MET* or *KRAS *amplification. Currently, several novel agents targeting *RET* fusions in NSCLC are being tested. TAS0953/HM06 and TPX-0046 are in phase 1/2, while RXDX-105, and BOS172738 are in phase 1 (Michelotti et al., 2022[58]). 

In 2020, a new inhibitor- Sotorasib was approved to treat NSCLC patients with *KRAS* mutation (Michelotti et al., 2022[58]). This drug is not a typical TKI inhibitor, but it is an inhibitor of the RAS GTPase family and targets a specific mutation, G12C, in the protein K-Ras. Concerning a high frequency of *KRA*S mutations, in many European countries, the test for *KRAS* as a biomarker in molecular diagnostics of NSCLC was recommended (Kerr et al., 2021[44]). *KRAS* mutated gene is a target of clinical trials NCT04504669 and NCT03101839 with antisense oligonucleotides (Bartolucci et al., 2022[5]). These molecules can be an emerging class of biotherapeutics for a new era of precision anti-cancer medicine.

The excellent response to inhibitors is observed not only in NSCLC patients with known driver mutations, like *EGFR*, *KRAS,* or *ALK* rearrangement. The results of Hu et al. (2019[32]), obtained for NSCLC Chinese patients indicate that the effect of EGFR-TKIs is also good in patients with mutations of the *HER2* gene (Michelotti et al., 2022[58]; Uy et al., 2022[86]) and germline *BRCA* mutations (Hu et al., 2019[32]). For *HER2*-altered NSCLC, clinical trials are conducted with antibody-drug conjugates, like T-DXd (Trastuzumab Deruxtecan), T-DM1 (Trastuzumab Emtansine), but also with TKIs, like Pyrotinib and Poziotinib (Uy et al., 2022[86]). In NSCLC patients, several clinical trials were also conducted with PARP inhibitors. For example, Olaparib versus placebo monotherapy was tested in a multicenter, randomized, controlled phase 2 (Fennel et al., 2022[24]). A clinical trial (phase 2) has been conducted with Niraparib plus immune checkpoint inhibitor Pembrolizumab (Ramalingam et al., 2022[71]). 

### Immune checkpoint inhibitors (ICIs)

PD-1/PD-L1 inhibitors are promising immunotherapeutic agents approved for many cancer types, including NSCLC (Jiang et al., 2019[38]). ICIs therapies give a chance to many NSCLC patients who do not show any driver gene mutations and are excluded from the targeted therapy. Based on the Chen et al. (2021[12]) meta-analysis, it can be found that ICIs give an excellent objective response rate and duration of response. The first ICI in NSCLC was approved in March 2015. It was Nivolumab, in the second-line treatment of advanced disease stage. A few months later, Pembrolizumab was approved, and in 2016 Atezolizumab as well (Jain et al., 2018[35]). Since 2017, Pembrolizumab can be applied as first-line systemic therapy for patients with PD-L1 expression in cancer cells >50 % or as a second-line systemic therapy after progression on first-line chemotherapy, with at least 1 % PD-L1 expression in tumor cells. High PD-L1 expression is also a predictive biomarker for Atezolizumab in monotherapy (Jassem et al., 2021[36]). Recent data led to the approval of combining a single ICI (Pembrolizumab) with chemotherapy (Gandhi et al., 2018[26]).

The association between PD-L1 expression and clinicopathological features is not clear. In the study of Pawelczyk et al. (2019[65]) conducted on 866 samples of NSCLC in TMA (tissue microarray) probes, it was found that the patients with low PD-L1 expression had prolonged overall survival compared to the group with high expression, but only in AC. According to the results of Pawelczyk et al., as well as of Igarashi et al. (2016[33]), higher expression of PD-L1 is noticed in G2 and G3 in AC patients, compared to G1. PD-L1 positivity is higher in males, smokers, positive blood vessels, and lymphatic invasion (Miyazawa et al. 2019[60]).

There was a concept that high tumor mutation burden (TMB) could be one of the predictors of the response to immunotherapy with ICIs. The higher the TMB level, the more new antigens are present and the more likely the immune system is to be activated against cancer cells (Dong et al., 2019[20]). Among NSCLC patients, those with *HER2* mutations show the lowest levels of PD-L1 expression. Hence, the effect of ICIs will be relatively weak (Vathiotis et al., 2021[87]). 

A pan-tumor retrospective analysis of participants with advanced solid tumors, including NSCLC, showed that TMB ≥175 mutations are associated with improvement in the effectiveness of Pembrolizumab monotherapy vs. chemotherapy (Cristescu et al., 2022[16]). However, large clinical trials did not confirm TMB as a useful predictive tool. In recent years, clinical trials have also been conducted on ICIs combinations (e.g., with chemotherapeutic agents and other ICIs) (Table 4(Tab. 4); References in Table 4: Paz-Ares et al., 2022[67], Pinto et al., 2019[69]; Zerdes et al., 2018[98]). Some clinical trials are focused on Ipilimumab, an anti-CTLA-4 ICI (Paz-Ares et al., 2022[67]). It was the first ICI approved in cancer immunotherapy (Tomasini et al., 2012[85]). However, this agent has shown limited efficacy as a single agent in lung cancer, compared to those obtained in melanoma. New Ipilimumab combinations with chemotherapeutic agents or other ICIs, like Nivolumab, show better effects (Lena et al., 2022[48]). 

### CAR-T cells 

CAR-T cell therapy can be another promising way for many NSCLC patients. T lymphocytes can be genetically modified to recognize antigens of NSCLC cells, like EGFR, PD-L1, mesothelin, mucin 1, and CEA (Qu et al., 2021[70]; Chen et al., 2022[10]). So far, the CAR-T cell procedure has been dedicated to hematological diseases, and the first CAR-T cell drugs, Kymriah and Yescarta, were approved in 2017 for patients with acute lymphoblastic leukemia and diffuse large B-cell lymphoma (Styczyński, 2020[82]). Many clinical trials are conducted with CAR-T cell therapy in solid tumors, including NSCLC (Barros et al., 2022[4]; Chen et al., 2022[10]). However, few severe obstacles exist in developing this strategy in solid tumors. Among them is a low level of tumor-specific antigens, T-cells infiltration, and high tumor microenvironment immunosuppression (Qu et al., 2021[70]). CAR-T cells are applicated intravenously. However, as seen in Table 5(Tab. 5) (References in Table 5: Chen et al., 2022[10]; Johnson et al., 2022[39]), impaired tumor vasculature and other tumor microenvironment (TME) features are responsible for the immunosuppressive influence on CAR-T cells (Johnson et al., 2022[39]). Despite these difficulties, many preclinical and clinical studies have been conducted with the aim to improve CAR-T cell access in solid tumors (Nguyen et al., 2022[61]). One of the clinical trials is the NCT04153799 study of CXCR5-modified EGFE chimeric antigen receptor autologous T-cells in EGFR-positive patients with advanced NSCLC (phase 1).

### miRNAs

Lung cancer cells show not only genetic but also epigenetic changes. There can be upregulation of oncogenic miRNAs, and downregulation of tumor suppressive miRNAs. Oncogenic miRNAs can be intensely involved in tumor growth, angiogenesis, epithelial-mesenchymal transition, or immune escape (Ahn et al., 2020[1]; Wang et al., 2019[89]). A great attention is paid to non-coding RNAs (miRNAs, circRNAs) in cancer stem cells responsible for cancer metastasis and drug resistance (Bryl et al., 2022[8]). Non-coding RNAs can be the new weapon in developing targeted therapies to combat cancer. For example, applying tumor suppressive miRNA mimetics or reducing oncogenic miRNAs in cancer cells seems to be a good perspective for a new therapeutic strategy. In recent years, several *in vivo* lung cancer models with miR-7, miR-34a, or miR-200c were tested (Ahn and Ko, 2020[1]). So far, two clinical trials with miRNAs have been conducted (Wang et al., 2019[89]). For example, in NCT02369198, a mimetic of miR16 was tested (phase 1). This potential drug consists of three components: 1) miR-16-based miRNA mimic, 2) drug delivery vehicle composed of non-living bacterial minicells (about 400 nm in size) which allow efficient drug packaging, 3) targeting moiety of nanoparticles to EGFR-expressing cancer cells with an anti-EGFR bispecific antibody (Kara et al., 2022[43]). 

## Conclusions

Significant correlations between driver gene alterations and therapeutic outcomes of NSCLC patients show that precise molecular diagnostics is crucial, and there is an urgent clinical need to applicate effective, targeted therapies. Hence, molecular diagnostic methods, like NGS, should become a daily practice. The unresolved problem in NSCLC patients under protein inhibitors' treatment is the acquired resistance to the drugs. Current studies should be concentrated on the mechanisms of the resistance and addressing them, as well as on looking for novel targets and tools. Therapies with ICIs, and their combinations with chemotherapy are also of great clinical interest. The novel treatment with CAR-T cell therapy or the application of miRNA-based drugs can also be a promising direction for NSCLC patients.

## Notes

Anna Grodzka and Agnieszka Knopik-Skrocka contributed equally as first author.

## Declaration

### Conflict of interest

Maciej Bryl COI: Honoraria, consulting or advisory role and travel, accommodations, expenses: Boehringer Ingelheim, Roche/Genentech, MSD, Bristol-Myers Squibb, AstraZeneca, Takeda, Novartis, Pfizer. Other authors declare no conflict of interest.

### Acknowledgments

The authors would like to thank Ms. Oliwia Piwocka from Radiobiology Laboratory, Department of Medical Physics, Greater Poland Cancer Center, Poznań, for professional language assistance.

## Figures and Tables

**Table 1:**
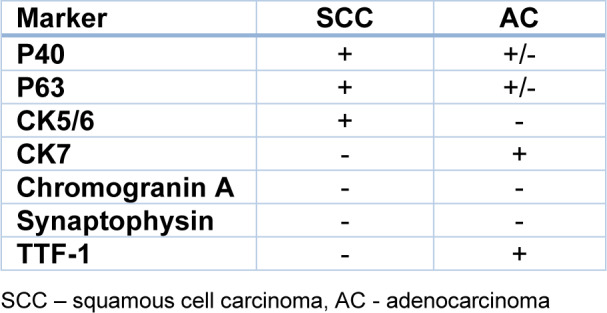
IHC markers used in NSCLC diagnostics (Zheng, 2016, changed)

**Table 2:**
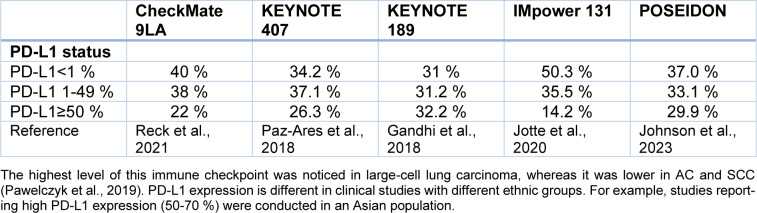
PD-L1 status in NSCLC patients according to five clinical trials

**Table 3:**
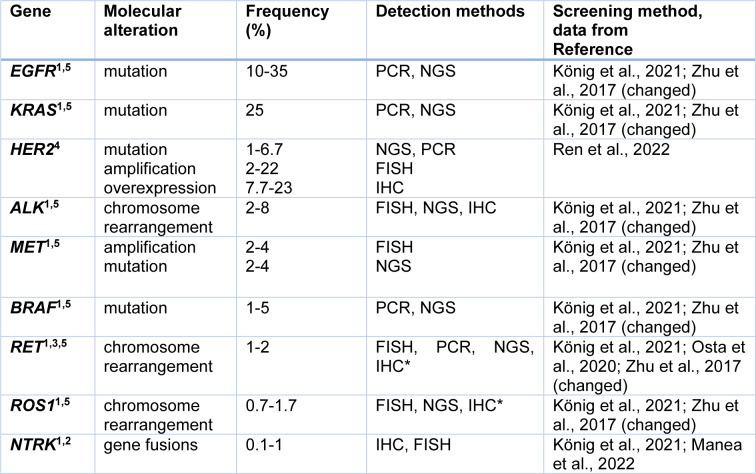
The main driver mutations in NSCLC and the methods used in their diagnostics

**Table 4:**
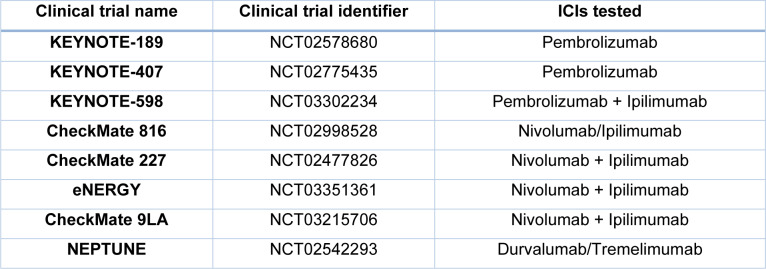
Phase 3 trials of ICIs or their combinations in NSCLC (Zerdes et al., 2018; Pinto et al., 2019; Paz-Ares et al., 2022, changed)

**Table 5:**
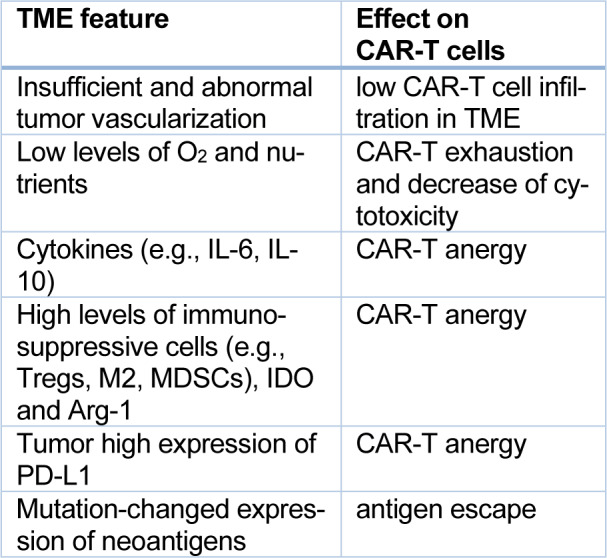
Immunosuppressive influence of TME on CAR-T cells (Chen et al., 2022; Johnson et al., 2022 changed)
